# Changes in French purchases of pulses during an FAO awareness campaign

**DOI:** 10.3389/fnut.2022.971868

**Published:** 2023-01-26

**Authors:** Ikpidi Badji, France Caillavet, Marie Josephe Amiot

**Affiliations:** ^1^Institut National de Recherche pour l'agriculture, l'alimentation et l'environnement (INRAE), Paris, France; ^2^Institut National de Recherche pour l'agriculture, l'alimentation et l'environnement, Montpellier, France

**Keywords:** awareness campaign, pulses purchases, Box-Cox double-hurdle model, pseudo-panel, nutritional guidelines, sustainability

## Abstract

**Background:**

Pulses can play a key role in a well-balanced diet and are now recognized for their health and sustainability benefits. However, consumption remains quite low, motivating promotion efforts such as the “International Year of Pulses” declared by the Food and Agriculture Organization (FAO) in 2016. The present study aims to evaluate the changes in the purchase of pulses before and after the FAO's awareness campaign promoting the consumption of pulses in France and investigate the potential differences across sub-populations.

**Methods:**

Purchase data come from Kantar Worldpanel 2014–2017. First, in order to understand demand for pulses, the influence of sociodemographic variables on the purchase of pulses in different forms (raw, processed, ultra-processed) is analyzed using a Box-Cox heteroskedastic double-hurdle model. Then, changes in purchasing before and after the FAO campaign were estimated using a two-way fixed-effects model, controlling for price and sociodemographic variables.

**Results:**

On that period, the purchasing of pulses increased by 8.4% overall. The increase was greater for younger participants (+11.8%), people living in urban areas with over 200,000 inhabitants (+8.4%), and lower-income households (+7.1%). The 8.4% increase observed indicated that there were gradual preference change in favor of pulses and the impact of the awareness campaign was to boost expenditure on pulses by a further 2%.

**Conclusion:**

The FAO campaign coincided with an increase in the purchasing of pulses and may have had an enhancing effect. However, consumption still remains below the level advised by dietary guidelines. There is a need for more public information and communication on the health and sustainability benefits of pulses, the consumption of which can be promoted through supply and education interventions.

**JEL codes:**

D12; Q18; I18.

## Introduction

### Health and sustainability benefits of pulse consumption

Pulses have been a focus of attention for several years in the developed economies, as a food group favorable to health and sustainability. According to the Food and Agriculture Organization (FAO), pulses have significant health benefits and are conducive to a well-balanced diet. Legumes/pulses^1^ are an interesting source of protein. They have a higher micronutrient density (high folate, iron, magnesium, potassium, and zinc content) than cereals, almost double the protein content, and a high lysine content (the main limiting amino acid in cereals), making them a perfect complement to cereals in diets with low amounts of animal products. In some countries, legumes/pulses are classified as protein foods, along with the traditional animal-protein group (meat, eggs, and fish) (1, 2). Beyond proteins, legumes are a dietary source of fiber and various micronutrients of interest (3, 4). Epidemiological studies have reported health benefits associated with consuming legumes.^2^ In addition, from an ecological point of view, the environmental cost of plant proteins is reduced compared to animal proteins, and pulses can be considered a sustainable alternative (9). Moreover, the benefits of meat consumption remain a topic of debate (9, 10). Dietary guidelines recommend the consuming pulses^3^,^4^ and limiting meat intake.^5^

In this context, pulse-based foods display several advantages at the consumption level. First, they are protein-rich and can be used as substitutes for meat in a healthy diet. Second, they have a lower cost, making these foods a cheap source of alternative proteins. Finally, they are easily storable, as they are mainly consumed in a non-perishable form (dry or canned). Despite these advantages, pulse consumption remains low in the Western diet (13). In France, the recommendation is “intake at least twice a week,” representing 200 g/week,^6^ far from what French dietary surveys register at the national level. The third National Food Consumption survey, INCA3,^7^ realized in 2014–2015, found an average consumption of 7.7 g/day in an adult sample, with only 14.7% of respondents reporting being consumers of pulses.^8^ In 2019, French consumers still considered pulses a traditional and old-fashioned food, and these were sometimes perceived as being part of low-budget diets (15), less fancy, food for vegetarians, requiring long preparation, not very well liked, or used as a side and not a main dish (16). Other studies have uncovered similar perceptions. In addition, consumption can be hindered by the presence of antinutrients causing digestive discomfort (17).

Few studies have investigated the demand for pulses in developed countries using household surveys.^9^ In the estimation of household demand systems, pulses and derived products are frequently included in large aggregated categories, such as fruits and vegetables or starchy foods (19). The substitution potential between animal products and more eco-friendly or plant-based alternatives remains the main issue. Studies investigate the potential reductions in meat consumption by assessing consumers' consumption of animal products associated with lower greenhouse gas emissions and, more rarely, of plant-based alternatives (20, 21). Few consumers replace meat by pulses to such a degree that a decrease in meat consumption is observed. Most consumers consume comparable amounts of meat but vary with respect to their acceptance of substitutions, and studies vary with respect to the options assessed: health information campaign, redesign of meal composition in convenient products, and meat substitutes. Consumers may also consider a change in meat portions (inducing the consumption of larger portions of legumes/pulses) or may lower their meat-eating frequency (with a higher intake frequency of legumes/pulses) (22). Different consumer groups favor different substitution options, indicating a role of sociodemographic characteristics in the demand for pulse-based products (23). According to a meta-analysis of meat-eating behavior, the most influential factors are gender, age, and socioeconomic status (SES) (24). Research has shown that young people are more open to flexitarianism, and that health concerns are associated with older age (in particular people 45–60 years of age), while younger consumers are more receptive to minimizing environmental impacts, as are people with a higher level of education (25, 26).

### The potential of information policies

The “mismatch” between the many benefits of pulses and the low levels of consumption has recently motivated promotion efforts. A lack of knowledge regarding the environmental issues associated with meat consumption (27–29) and the possibilities for the substitution of meat by plant protein sources (30) are cited as reasons for the mismatch. However, public awareness was raised through the dissemination of scientific information regarding meat consumption,^10^ and official promotion was undertaken through an international FAO campaign declaring 2016 as the International Year of Pulses (IYP) (32).^11^ In France, 24 national and regional events took place in 2016, promoted on the Ministry of Agriculture's website and relayed by the media. The campaign raised awareness among different food-system actors, including policymakers, pulse producers, processors, and traders, restaurant and catering operators, health and nutrition practitioners, and end-users such as schoolchildren. The general public was the focus of an information and educational campaign based on mass media and face-to-face events (cooking demonstrations, exhibitions, and museum displays—in particular, at the SIAL^12^ (Salon International de l'Alimentation) international food exhibition held in Paris. Recently, a study found that the message regarding the environmental benefits of pulses and crop diversification tended to have a greater impact on intentions to purchase lentils than information about their nutritional benefits (34). Firms understood the new opportunities: they began displaying environmental claims on pulse packaging (35) and invested in innovations based on pulses. New and exotic products emerged on the market, such as prepared snacks (hummus and other chickpea-based foods) or dishes (lentil- or pea-based), pasta made with pulse flour, soy preparations, and many combinations of pulses and other ingredients (36). A two-tier market thus appeared, with both a traditional segment of pulses (unprocessed raw products, canned, or less processed, such as preserved or frozen) and an innovative one including ultra-processed products, and in particular, meatless substitutes (soy steaks, vegan sausages, etc.). Firms anticipated this surge—by 2016, large agri-food companies had started acquiring successful meat-substitute firms around the world (37).

Several methods have been developed to evaluate the impact of information policies. Contrary to the abundant literature on trials enrolling control groups to evaluate the impact of a specific intervention, the FAO campaign focused on here involved global exposure, without the possibility of a control/non-exposed group. Moreover, we cannot rely on an estimation of variation in the degree of exposure of the population to the campaign (38). Some previous works have also faced these issues. A first approach, used by Capacci and Mazzocchi (39) in a study on the UK's 5-a-day promotion of fruit and vegetables, involves estimating the counterfactual, i.e., the outcome expected in the absence of an information campaign. The authors mentioned measured the average treatment effect on the exposed subjects, corresponding to the entire population. Another approach successfully used by Shankar et al. (40) in a study of a campaign aiming to limit salt consumption involved (i) a fixed effects model to analyze the trend in salt intake over the period considered and (ii) a two-way fixed-effects model to estimate group-specific responses to the campaign, where each cross-sectional unit was considered as its own control group. In both works, the impact of the health information campaigns was found to be positive. One study also evaluated the impact of a campaign across income quartiles (39). The campaign was found to be more effective for those in the 3rd income quartile and therefore somewhat regressive in terms of reducing nutritional inequalities. Information campaigns are one of the tools that are frequently used to implement public food policy (for instance, by the French Nutritional and Health Program). However, two issues can affect their efficiency. First, public budgets for safe and nutritious foods appear limited compared to the large budgets earmarked by the agroindustry to advertise “junk food.” Second, information is disproportionately well received by more educated consumers, inducing nutritional and health inequalities (41, 42). Nevertheless, as this policy tool can be used at the meta-level of countries when international organizations such as FAO get involved, it can have a significant impact.

In this context, our study aims to analyze the changes in pulse purchasing before and after the FAO campaign and discuss the possible effects of this awareness policy on pulse consumption in France, with particular reference to different sub-populations. To this aim, we consider a fixed-effects model to estimate the changes in purchasing concomitant with the FAO campaign. Our data consist in purchases for food-at-home. In a first step, as the demand for pulses has not been the focus of many works and remains poorly understood, we analyze the influence of sociodemographic factors on the purchase of pulses using a Box-Cox double-hurdle model. In a second step, we analyze the changes in purchasing associated with the 2016 FAO campaign, at the population level and across sociodemographic groups, to test whether it might have had a regressive effect by affecting population subgroups differently, which could deepen nutritional inequalities.

The remainder of the paper is organized as follows. Section Methods describes the data and methodology. Section Results presents the results. Section Discussion discusses the results and policy implications. Section Conclusions offers some concluding remarks.

## Methods

### Data

The data employed here are from Kantar Worldpanel surveys conducted in 2014, 2015, 2016, and 2017. These surveys were administered to over 20,000 households, which reported their weekly food acquisition. All participating households registered grocery purchases through the use of barcode scanning. Then, a group of households (half of the participating households) was asked to also specifically report the purchases of fresh products (meat, fish, fruit, and vegetables). These purchasing data provide an inventory (in quantity and expenditure) of the French population's consumption of food-at-home. However, these data do not provide information on food-away-from-home or self-produced food consumption (for example, from a home garden), thus they represent around 80% of total consumption.^13^

For our study, we selected the group of households with complete information on purchases, i.e., who reported both their grocery purchases and their fresh produce acquisition (over 12,000 households). It is worth noting that the Kantar data distinguish 13 periods of 4 weeks each throughout the year. Therefore, we retained households that reported their food acquisition for at least 1 week. We excluded households that did not communicate information about sociodemographic and economic variables (such as income or education level). Consequently, our sample includes 10,914 households in 2014, 11,074 households in 2015, 11,031 households in 2016, and 10,764 households in 2017. Each database is considered as a cross-section.^14^ We selected a set of products including pulses in different forms, including raw and subject to various preparations: dried (lentils, beans, flageolet beans, split peas, and chickpeas), canned (flageolet beans, white and red beans, lentils, and chickpeas), frozen (flageolet beans), preparations/recipes (falafel, tofu, sausages with lentils, soy steaks, and other meat substitutes), desserts made of soy, soy beverages, and soy ingredients.

The dependent variable used in our analysis was the annual purchase of pulse-based products at the individual level. We obtained this by cumulating the period quantities registered in the Kantar database at the household level for each year of study. We then divided these quantities by the number of persons in the household. Therefore, the purchase of pulse-based products in kg/year/pers is expressed as follows:


$$\begin{array}{l} q\frac{Kg}{Year}/pers=\frac{quantity_{{kg}^{*}}\frac{52}{Nw}}{N}, \end{array}$$

where *Nw* is the number of weeks of food purchases in a given year and *N* is the number of persons in the household.

**Figure 1:**
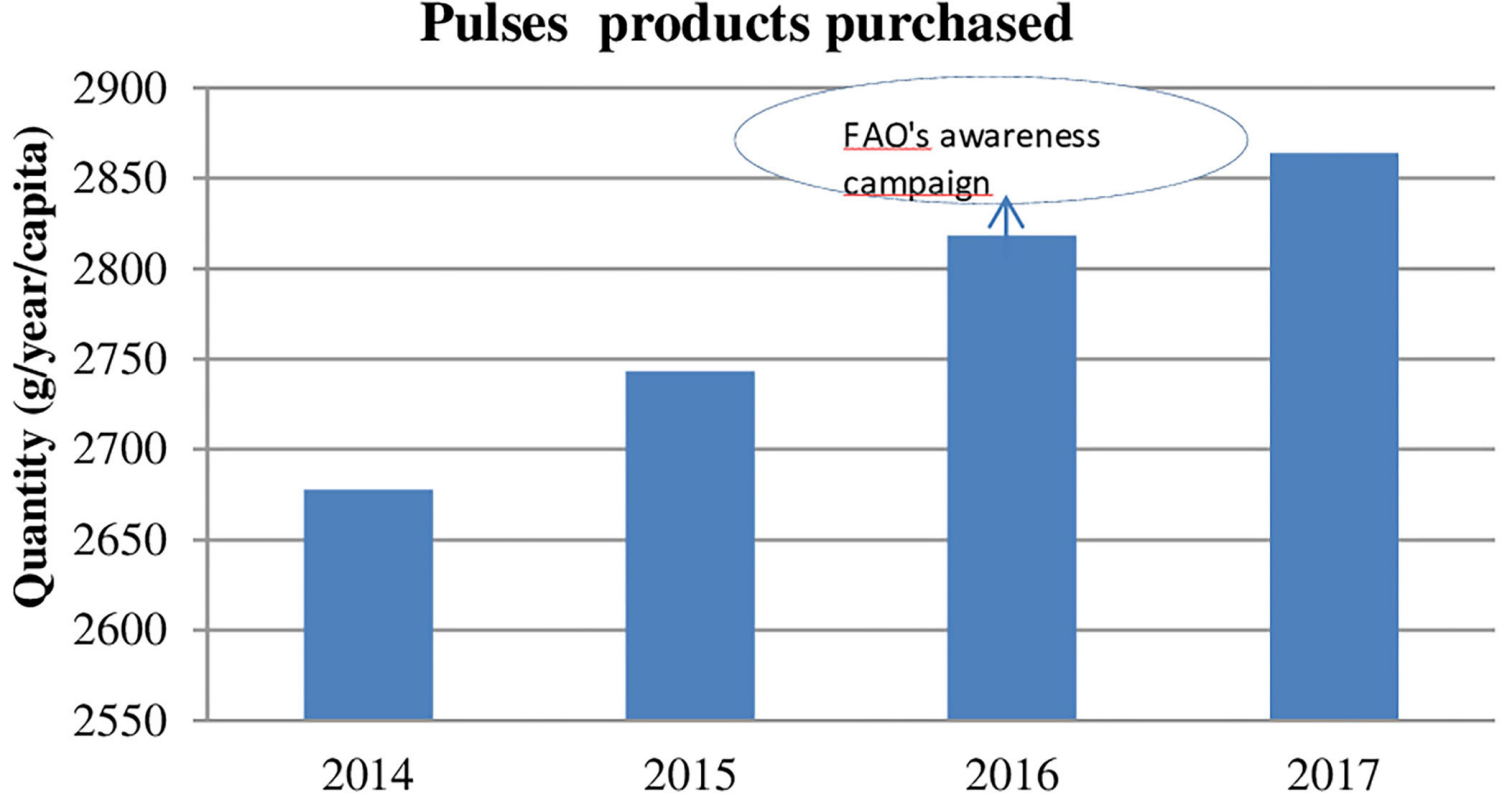
Evolution of purchased quantity of pulses products (g/year/cap), 2014–2017. Source: Kantar Worldpanel 2014–2017.

**Figure 2:**
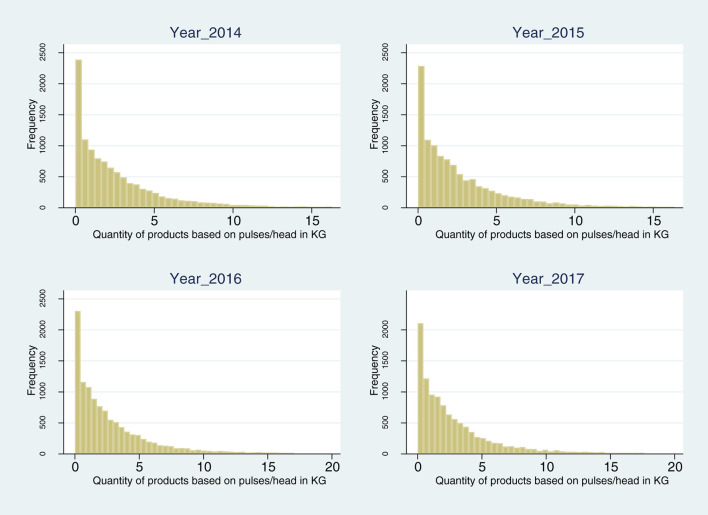
Proportion of zero purchases in the sample 2014–2015. Source: Kantar Worldpanel 2014–2017.

The purchasing of pulse products increased by 6.9% between 2014 and 2017 (Figure 1; 2.68 kg/year per capita in 2014 and 2.86 kg/year in 2017, Appendix A). A large majority of consumers (84.9–88.4%) bought pulse-based products (Figure 2; Appendix A). Taking into account only pulse consumers, quantities per capita represented 3.24 kg in 2017. It must be noted that, in contrast to the purchases by the total sample, purchases attributed only to pulse consumers show stagnation between 2016 and 2017, at the time of the FAO awareness campaign. Relative to quantity, expenditure per capita showed a greater increase of 12.2% (Appendix A), meaning an increasing average unadjusted unit value,^15^ from 2.77 to 2.94 €/kg. The market broadened throughout the period under analysis. This can first be captured in terms of the increase in the diversity of products, observable in the 16.3% increase in the number of different barcodes in the Kantar categories “pulses” and “preparations from pulses” (2,049 items in 2015 and 2,383 in 2017). To capture the polarization of the market, we separated the pulse-based products into two segments: less-processed products (unprocessed and preserved, i.e., traditional consumption) and ultra-processed products (preparations from pulses, including meat substitutes). The proportion of purchasers of both categories increased across the period under investigation (73.2–77.2% for less-processed products, or LPs, and 60.0–63.6% for ultra-processed products, ULPs), with a stable repartition of quantities and close proportions between these two segments: LPs represented 59.3% of the market in 2014 and 58.6% in 2017. However, average prices (unadjusted unit values) were quite different. Not only was the price difference between LPs and ULPs in a range of one to two, but the price increase was also higher, in a range of one to five, with an increase of 2.7% for LPs and 14.0% for ULPs (Appendix A). The increase in consumption over this period may be attributable to various drivers, such as greater awareness (due in part to the FAO campaign), greater diversity in supply, mainly driven by consumer demand and firm restructuring (36, 37), and the flexitarian behavior of consumers with a growing acceptance of alternatives to meat (20) and concern for animal welfare. Heterogeneity in purchasing is observed according to sociodemographic characteristics. In particular, higher quantities are purchased by people with an education level lower than the baccalaureate diploma, those 65 years and over, and in the southern region (Appendix B).

### Econometric methodology

#### Estimation of the relationship between sociodemographic characteristics and pulse purchases using a Box-Cox double-hurdle model

Our aim is to identify the sociodemographic factors associated with the purchase of pulse products. Linear regression models are generally inappropriate for analyzing such data since some households may not purchase pulses in a given year, i.e., the presence of zeros. The Tobit model (45) is often used when a dependent variable is zero for a part of the population but positive (and with different outcomes) for the rest of the population. Here, we specify the Tobit model as follows:


(1) $$\begin{array}{l} y_{h}^{*}=\;x_{h\;}\beta \;+\varepsilon_{h}\;\forall \;h=\;1,\ldots .N\\ \;y_{h}=\;\{\begin{array}{c} y_{h}^{*}\;if\;y_{h}^{*}>0\\ 0\;if\;y_{h}^{*}\leq 0, \end{array} \end{array}$$

where $y_{h}^{*}$ is the latent variable and *y*_*h*_ is the quantity of pulse products purchased. $x_{h}=\left(x_{h}^{1}\ldots x_{h}^{K}\right)$ is a vector of explanatory variables, $\beta ={\left(\beta_{1}\ldots \beta_{K}\right)}^{\prime }$ is a vector of unknown coefficients, and $\varepsilon_{h}^{\prime }s$ are independent, identical, and normally distributed random variables with a mean of zero and variance σ^2^. The estimation procedure used was the maximum likelihood method.

The Tobit model assumes that households simultaneously decide to purchase pulse products and what quantity to purchase. In other words, this model assumes that the sign of a given determinant's effect will be the same for both the probability of purchasing pulse products and the quantity purchased.

The double-hurdle model by Cragg (46)^16^ is an alternative method that supposes sequential behavior. In a first step, households decide whether to purchase pulse products. The second step models the quantity they will buy, conditional on the first decision. Therefore, in Cragg's model, the decision to participate in the market (i.e., purchase pulse products) and the level of participation (i.e., the quantity of pulse products purchased) are determined by two separate mechanisms (47). The participation (P) equation is as follows:


(2) $$\begin{array}{l} P_{h}=1\;if\;P_{h}^{*}>0\;and\;0\;if\;P_{h}^{*}\leq \;0;\\ \;P_{h}^{*}=\;\alpha z_{h}+u_{h}\;u_{h}\sim N\left(0,\;1\right), \end{array}$$

where P^*^ is a latent participation variable that takes a value of 1 if the household purchased pulse products and 0 otherwise; *z* is a vector of household characteristics; and α is a vector of parameters. The level of participation (Y) equation is follows:


(3) $$\{\begin{array}{l} y_{h}=\text{​​​​​​​​​​​​​​​​ }y_{h}^{*}\;if\;y_{h}^{*}>0\;and\;P_{h}^{*}>0\\ y_{h}=\text{ ​​​​​​​​​​​​​​​​​​​​​​​​​​​​​​​​​​​​​​​​​​​​​​​​​}0\;otherwise\\ y_{h}^{*}=\;x_{h\;}\beta +\;\varepsilon_{h}\;\varepsilon_{h}\sim N\left(0,\;\sigma^{2}\right),\; \end{array}$$

where *y*_*h*_ is the quantity of pulse products purchased, *x* is a vector of household characteristics, β is a vector of parameters, and *u*_*h*_ and ε_*h*_ are independently distributed. The estimation procedure used was the maximum likelihood method. To test which model—the Tobit or Cragg model—best identifies the determinants of pulse products purchases, a likelihood ratio (LR) test was conducted (Appendix C) (48).

##### Specification issues

According to Arabmazar and Schmidt (49), the standard double-hurdle model built on the assumption of normality of the error may be inconsistent when this normality assumption does not hold. Thus, we tested normality by conducting the Doornik–Hansen (50) tests. The test indicated that we could reject that the residuals were normally distributed (Appendix C). One way to manage the non-normality of the error is to apply a Box-Cox transformation to the dependent variable (51–53), such as


(4) $$\begin{array}{l} y^{T}=\;\frac{y^{\lambda }-1}{\lambda }, \end{array}$$

where *y*^*T*^ corresponds to the transformed variable and λ is the transformation parameter to be estimated, which is between 0 and 1. To take into account the presence of heteroskedasticity,^17^ or the variation in the variance values across the observations, we specified the variance of the error terms as a function of a set of variables. The standard deviation can be written as follows


(5) $$\begin{array}{l} \sigma_{h}=exp\left(\;\gamma M_{h}^{{}^{\prime }}\right), \end{array}$$

where *M*_*h*_ is a vector of the variables^18^ and γ is a vector of the coefficients (54, 55).

#### Estimation of changes in pulse purchasing over the awareness campaign period using a two-way fixed-effects model

##### Estimation strategy

To more accurately evaluate the changes in purchase behavior during the awareness campaign period, the standard procedure would be to define a treated and a control group and to apply an estimation method such as difference-in-difference. In our case, the entire population was exposed to the awareness campaign considered as the treatment, and there is no possibility of distinguishing a control group from the treated one. Therefore, we follow the strategy proposed by Lee and Jones (56) and used also by Shankar et al. (40). This method consists in using panel data to estimate

1- A one-way fixed-effects model including a variable that identifies the change in household quantities of pulse products purchased over time, after controlling for observable factors that may affect the outcome. This is described as follows:

(6) $$\begin{array}{l} Y_{ht}=\alpha +\beta X_{ht}+\rho D_{t}+\vartheta_{h}+\varepsilon_{ht} \end{array}$$

where *Y*_*ht*_ is the logarithm of quantity of pulse products purchased per/head for household *h* at time *t*.

*X*_*ht*_ stands for a vector of sociodemographic and economic variables. *D*_*t*_ is a dummy variable taking a value of 0 and 1 for the pre-campaign and post-campaign periods, respectively. ϑ_*h*_ represents individual effects, and ε_*ht*_ is the residual.

2- A two-way fixed-effects model used to separately estimate the individual fixed-effects before and after the awareness campaign. This is described as follows:

(7) $$\begin{array}{l} Y_{ht}^{0}=\alpha^{0}+\beta^{0}X_{ht}^{0}+\tau^{0}\delta_{t}^{0}+\vartheta_{h}^{0}+\varepsilon_{ht}^{0},\\ Y_{ht}^{1}=\alpha^{1}+\beta^{1}X_{ht}^{1}+\tau^{1}\delta_{t}^{1}+\vartheta_{h}^{1}+\varepsilon_{ht}^{1}.\; \end{array}$$

The superscripts pertain, respectively, to pre- (noted 0) and post-campaign (noted 1), and δ_*t*_ represents time dummies. With this method, each value of the individual effect is predicted and then used to compute the *full* individual fixed effect, which is equal to the mean of time fixed effects plus individual fixed effects. The differences between the “pre-campaign” full individual fixed effects and the “post-campaign” ones indicate the individual response in this period of the awareness campaign. In other words, holding other observable variables constant, they tell us how the pulse-product purchasing of each household changed in the post-campaign period.

Since our data are cross-sectional, we used them to establish a pseudo-panel. The idea is to identify households belonging to the same cohort^19^ and to monitor the mean behavior of the cohorts established (57). In total, two types of problem tend to be generated by estimations from pseudo-panels. The first concerns measurement errors for the different variables, which can lead to estimation biases. The model variables are not directly observed but, rather, the mean values are calculated using survey data. Nevertheless, these are close to their true values when there are a large number of individuals in the cohort. Verbeek and Nijman (58, 59) showed that measurement errors and estimation biases are negligible if the size of cohorts reaches 100. However, establishing large cohorts involves reducing the number of observations used (here, the number of cohorts) across a given sample, leading to less precise estimations. Reducing the number of cohorts can also increase the heterogeneity of individuals in a single unit and, therefore, increase the variance of estimators, making them less effective. A compromise needs to be found between the cohorts large enough to limit measurement errors, sufficiently homogeneous cohorts, and sufficient observations to obtain adequately precise estimators. We have four cross-sectional databases spanning 2 years (2014 and 2015) before the awareness campaign and 2 years (2016 and 2017) after. Each “pre-campaign” and “post-campaign” period includes 8 quarters. Each dataset contains more than 10,000 households for 13 periods of 4 weeks (52 weeks of the calendar year). We used the sum of those 13 purchasing periods in order to have quarterly purchasing information for each household.^20^ We defined our cohorts using two variables: the “year of birth” of the reference person in the household and “county size,” composed of three levels (rural area with fewer than 2,000 inhabitants, urban area with 2,000 to 199,999 inhabitants, urban area of 200,000 inhabitants or more). Thereby, we formed 165 annual cohorts observed at minimum over 8 quarters and at maximum over 16 quarters (4 years^*^4 quarters). The first three cohorts include the heads of household born in 1936 and living, respectively, in the three types of counties mentioned above. The last three cohorts include the heads of household born in 1990 and living, respectively, in the three types of counties mentioned. Our pseudo-panel includes 2,592 observations (study sample). Not all cohorts are observed in each survey, and the mean size of the observed cohorts is 64 individuals.

##### Robustness check

Although our pseudo-panel provides a large number of cohorts, which is important to obtain precise estimations, the number of observations per cohort is a little less than recommended. For this reason, we challenge the estimation from this pseudo-panel with a robustness check consisting of using the same sample and constructing another pseudo-panel—pseudo-panel 2—with cohorts of, on average, 100 individuals or more (59). Pseudo-panel 2 was constructed similarly, based on “year of birth” and “county size” variables (Appendix H). This pseudo-panel is composed of 84 biannual cohorts observed at minimum over 8 quarters and at maximum over 16 quarters. It includes 1,320 observations, and each cohort includes 127 individuals, on average. Considering the pseudo-panel nature of the data, equations take the following form:


(8) $$\begin{array}{l} Y_{ct}=\alpha +\beta X_{ct}+\rho D_{t}+\vartheta_{c}+\varepsilon_{ct}, \end{array}$$

(9) $$\begin{array}{l} Y_{ct}^{0}=\alpha^{0}+\beta^{0}X_{ct}^{0}+\tau^{0}\delta_{t}^{0}+\vartheta_{c}^{0}+\varepsilon_{ct}^{0},\\ Y_{ct}^{1}=\alpha^{1}+\beta^{1}X_{ct}^{1}+\tau^{1}\delta_{t}^{1}+\vartheta_{c}^{1}+\varepsilon_{ct}^{1}, \end{array}$$

where the household index *h* has been replaced by the cohort index *c*. As in equations (6) and (7), *X*_*ct*_ stands for a vector of sociodemographic economic variables: age, access to an orchard,^21^ access to a vegetable garden, education level, region of residence, body mass index, income class, and the logarithm of the price of pulse-based products.

##### Independent variables

Price is a key factor; however, the database does not provide a price variable. We computed the unit value of pulse-based products as the ratio between expenditure and the quantity purchased and adjusted the unit value for quality by following the procedure of Crawford et al. (60), who attributed variation in actual price to spatial and time differences only.

Other independent variables included were the socioeconomic and demographic characteristics of the household. These include the characteristics of the participant in the survey, namely, age, level of education, and socioprofessional category, which are considered major individual drivers of dietary patterns in protein intake (24). Our age variable has 3 levels, distinguishing 2 potentially active phases (18–44 and 45–64 years) from the retirement period (from 65 years). Our educational variable has 4 levels, distinguishing survey participants with less than post-secondary qualifications (around one third of the sample), post-secondary qualifications, and 2 categories above that level: those who completed up to 3 years of university and those who completed more than 3 years of university education (this latter group represents around 20% of the sample, depending on the year). The socioprofessional category of survey participants is expressed in 5 categories to differentiate labor status and consumption patterns: farmer, self-employed, employee/manual worker, associated professional, senior executive, and student/unemployed. Body mass index (BMI) was introduced as a health-related variable with 5 levels.^22^ Inverse associations were found between a higher consumption of pulses and a lower risk of overweight/obesity (7). At the level of the household, variables include household income, which was corrected by consumption units (CU) according to the OECD-modified scale. By taking into account demographic variation during the life cycle, the measure of income per consumption unit allows for a comparison of the incomes of households of different sizes and composition. Our income/CU variable has 4 levels: lower than poverty line; from poverty line to median income; from median income to 7th decile; 7th decile and over. We also introduced variables describing the household-specific access to fruit, vegetables, and pulses from self-production in orchards or vegetable gardens. Finally, we added location variables such as the region, distinguishing South from North based on the different pulse production capacities and consumption patterns. We also introduced county size, distinguishing rural areas from less and more urbanized areas (respectively, 2,000–199,999 inhabitants and 200,000 inhabitants and over). Descriptive statistics are presented in Table 1.

**Table 1:** Descriptive characteristics of the study population.

	**2014**		**2015**		**2016**		**2017**	
**Variables**	***N* = 10914**	**%**	***N* = 11074**	**%**	***N* = 11301**	**%**	***N* = 10764**	**%**
**Age Group**								
18–44 yrs	5,638	51.66	5,752	51.94	5,517	48.82	5,123	47.60
45–64 yrs	3,511	32.17	3,550	32.06	3,910	34.60	3,788	35.19
65 yrs and +	1,765	16.17	1,772	16.00	1,873	16.58	1,852	17.21
**Education level**								
< Post-secondary qualifications	3,112	28.51	3,073	27.75	3,131	27.71	2,954	27.44
Post-secondary qualifications	2,879	26.38	2,967	26.79	3,089	27.33	2,952	27.42
1st, 2nd, 3rd year university	2,531	23.19	2,557	23.09	2,612	23.11	2,485	23.09
Bachelor's degree and +	2,392	21.92	2,477	22.37	2,469	21.85	2,373	22.05
**Monthly income** €**/CU**								
< poverty line	1,839	16.85	1,895	17.11	1,885	16.68	1,728	16.05
Poverty line to median income	4,145	37.98	4,181	37.76	4,303	38.08	4,788	44.48
Median income to 7th decile	2,341	21.45	2,363	21.34	2,419	21.41	1,616	15.01
> 7th decile	2,589	23.72	2,635	23.79	2,694	23.84	2,632	24.45
**Body Mass Index**								
Thinness: BMI < 18.5 kg/M2	400	3.67	401	3.62	400	3.54	368	3.42
Normal weight: 18.5 ≤ BMI < 25 kg/M2	5,807	53.21	5,820	52.56	5,859	51.84	5,512	51.21
Overweight: 25 ≤ BMI < 30 kg/M2	2,884	26.42	2,985	26.96	3,102	27.45	2,985	27.73
Moderate obesity: 30 ≤ BMI < 35 kg/M2	1,128	10.34	1,127	10.18	1,192	10.55	1,208	11.22
Severe and morbid obesity: BMI > 35 kg/M2	497	4.55	520	4.70	532	4.71	499	4.64
No answer	198	1.81	221	2.00	216	1.91	192	1.78
**County Size**								
Urban area- 2,000–199,999 inhabitants	3,890	35.64	3,961	35.77	4,056	35.89	3,893	36.17
Urban area 200,000 inhabitants+ and Paris	3,790	34.73	3,722	33.61	3,686	32.62	3,415	31.73
Rural area	3234	29.63	3,391	30.62	3,559	31.49	3,456	32.11
**Region of residence**								
North	7,068	64.76	7,138	64.46	7,231	63.99	6,879	63.91
South	3,846	35.24	3,936	35.54	4,070	36.01	3,885	36.09
**Orchard owner**								
No	6,024	55.20	6,053	54.66	6,040	53.45	5,722	53.16
Yes	4,890	44.80	5,021	45.34	5,261	46.55	5,042	46.84
**Vegetables garden**								
No	6,509	59.64	6,493	58.63	6,572	58.15	6,149	57.13
Yes	4,405	40.36	4,581	41.37	4,729	41.85	4,615	42.87
**Socio-professional category**								
Farmer	55	0.50	55	0.50	60	0.53	51	0.47
Senior executive	729	6.68	741	6.69	718	6.35	726	6.74
Student/Unemployed person	927	8.49	923	8.33	837	7.41	773	7.18
Employee/Manual worker	5,339	48.92	5,412	48.87	5,525	48.89	5,264	48.90
Associated professionals	3,480	31.89	3,534	31.91	3,711	32.84	3,533	32.82
Self-employed	384	3.52	409	3.69	450	3.98	417	3.87

In the estimation of the Box-Cox double-hurdle model for pulse product purchases, the sociodemographics introduced could differ for each decision, separating the different determinants of the probability of purchasing from those of the decision regarding the amount purchased. Therefore, the decision to buy a specific product, a behavior related to taste, is separated from the decision about how much to buy, which could be related to health or sustainability behaviors. In the participation equation (decision to purchase), sociodemographic characteristics (age, BMI of the participant), SES (household income, socioprofessional status/education of the participant), self-production (orchard, vegetable garden), and location (region) were introduced. In the equation related to the decision regarding the yearly amount purchased, sociodemographic characteristics (age, BMI of the participant), SES (socioprofessional status/education of the participant), and location (region, county size) were used. For each equation, we estimated two specifications depending on the SES variables included. When simultaneously using SES variables within the same equation, problems of collinearity can occur. Specification 1 included income and socioprofessional status (excluding education). Specification 2 included income and education (excluding the socioprofessional status). In the fixed-effects model explaining the change in purchases concomitant with the FAO campaign, we introduced the price of pulses (corrected unit value) and all household characteristics described above. Concerning SES status, we chose Specification 2, which includes education, as knowledge (for which education level is considered a proxy) is assumed to be an important driver of receptiveness to health information (41).

## Results

According to the specification tests (Appendix C), the Box-Cox model was the best model, and we discuss these results in the following.

### What is the profile of consumers of pulse products?

The Box-Cox heteroskedastic double-hurdle model was estimated for 2015 and 2017, which correspond to the pre- and post-campaign years, respectively (Specification 1 is presented in Appendix D, Specification 2 in Table 2). Comparable estimations were performed for LPs (Appendix E) and ULPs (Appendix F).

**Table 2:** Associations of sociodemographic and economic variables with purchases of products based on pulses 2015 and 2017 Box-Cox heteroscedastic double-hurdle model.

	**Year 2015**		**Year 2017**	
	**Specification 2**		**Specification 2**	
**Variables**	**Participation**	***Y* ^*T*^**	**Participation**	***Y* ^*T*^**
**Age**				
18–44 years	−0.33***	−0.26***	−0.22***	−0.30***
	(0.037)	(0.029)	(0.038)	(0.028)
45–64 years	Ref	Ref	Ref	Ref
65 years +	−0.05	0.12***	−0.06	0.09**
	(0.050)	(0.035)	(0.050)	(0.035)
**County size**				
rural		−0.00		0.04
		(0.029)		(0.029)
Urban area - from 2,000 to 199,999 inhabitants		Ref		Ref
Urban area of 200,000 inhabitants+ and Paris		−0.05		0.03
		(0.029)		(0.030)
**Region of residence**				
North	0.02	−0.07***	0.00	−0.05*
	(0.032)	(0.025)	(0.033)	(0.025)
South	Ref	Ref	Ref	Ref
**Monthly income** €**/CU**				
< Poverty line	0.11**	−0.14***	0.05	−0.03
	(0.049)	(0.040)	(0.058)	(0.045)
Poverty line to median income	0.18***	−0.16***	0.06	−0.02
	(0.041)	(0.033)	(0.048)	(0.037)
Median income to 7th decile	Ref	Ref	Ref	Ref
>7th decile	0.01	−0.08**	0.02	−0.01
	(0.044)	(0.036)	(0.052)	(0.041)
**Body mass index**				
Thinness : BMI < 18.5 kg/M2	−0.04	0.09	0.06	−0.02
	(0.080)	(0.065)	(0.091)	(0.068)
Normal weight: 18.5 ≤ BMI < 25 kg/M2	Ref	Ref	Ref	Ref
Overweight : 25 ≤ BMI < 30 kg/M2	−0.13***	0.03	−0.06*	−0.00
	(0.036)	(0.028)	(0.038)	(0.029)
Moderate obesity: 30 ≤ BMI < 35 kg/M2	−0.00	0.05	−0.10*	0.03
	(0.053)	(0.040)	(0.052)	(0.040)
Severe and morbid obesity: > 35 kg/M2	−0.15**	0.13**	−0.10	0.05
	(0.070)	(0.058)	(0.075)	(0.058)
No answer	−0.05	0.03	0.14	−0.12
	(0.109)	(0.085)	(0.131)	(0.089)
**Orchard owner**				
Yes	0.14***		0.15***	
	(0.036)		(0.037)	
No	Ref		Ref	
**Vegetables production at home**				
Yes	0.13***		0.11***	
	(0.036)		(0.038)	
No	Ref		Ref	
**Education level**				
< Post-secondary qualifications	−0.02	0.10***	0.09**	0.11***
	(0.044)	(0.033)	(0.046)	(0.033)
Post-secondary qualifications	Ref	Ref	Ref	Ref
1st, 2nd, 3rd year university	−0.05	−0.10***	0.02	−0.10***
	(0.043)	(0.034)	(0.045)	(0.034)
Bachelor's degree and +	−0.08*	−0.15***	0.02	−0.10***
	(0.045)	(0.036)	(0.047)	(0.037)
Constant	1.16***	1.14***	1.16***	1.01***
	(0.056)	(0.045)	(0.063)	(0.048)
Box-Cox Parameter (λ)		0.19***		0.18***
		(0.009)		(0.009)
Log likelihood	−24,606.52		−24,194.85	
Observations	11,074	11,074	10,764	10,764

Standard errors in parentheses: ***p < 0.01, **p < 0.05, *p < 0.10. Specification 2 includes all variables except socioprofessional status variable. Specification 1 is in Appendix D.

#### 2015 purchases

The *probability of purchasing pulse products* (Specification 2) was associated with age, with a lower probability of purchase observed for younger panelists (at the global sample level) compared to individuals in the 45- to 64-year age group, and a higher probability of purchase for senior participants (65 years and over) when it comes to ULPs. Regarding education, we observed a lower probability of purchase for those holding a bachelor degree and over (with a level over 3 years of university) compared to those with post-secondary education, a difference significant at the 10% level. As for BMI, both an overweight and a severe/morbid obesity status were statistically associated with a lower probability of purchasing pulses (compared to those with a normal weight). Lower-income categories (under the poverty line and under the median income) had a higher probability of purchase compared to those with a higher-than-median income). Regarding socioprofessional status, farmers had a lower probability of purchase at the global level and for LPs (compared to employees/workers, in Specification 1; Appendix D). We observed a positive association between access to an orchard or a vegetable garden and the purchase of pulse products. Depending on the degree of processing of pulse products, we found similar associations but also some different effects, such as a negative association in the northern region (compared to the South) with LPs purchases.

The *quantity of pulse-based products purchased* showed a negative association with younger panelists and a positive one with older ones (reference: median category of 45–64 years of age). A severe obesity status was associated with greater quantities of pulse products purchased, as were the categories of overweight and moderate and severe obesity for LPs specifically. For ULPs, only the thin category showed a relationship to purchasing quantity. Income was negatively associated with the low-to-median category in both specifications and also to the highest category (reference: median income to the 7th decile). This latter effect was not found in the case of LPs. In Specification 1, categories of socioprofessional status such as associated professionals and senior executive showed a negative effect (reference: employees/workers). This latter effect was not observed for ULPs, however. Concerning education (Specification 2), a positive association was observed with the lowest level and a negative one with the level of 1–3 years of university (reference: post-secondary qualification), while only the highest level was significantly negative for ULPs. We also found a negative association of the northern region with quantities purchased, at the global level and for LPs. For ULPs, we observed a negative association with urban areas with over 200,000 inhabitants (reference 2,000–199,999 inhabitants).

It is interesting to note that for several characteristics, opposite effects were observed for the probability of purchase and the quantities purchased. Concerning income, the two categories of poorer households (under the median income) had a higher probability of purchasing LPs, but they purchased a lower amount. For ULPs, this effect was found only for the poverty-to-median income level. Concerning BMI, participants with severe and morbid obesity had a lower probability of purchasing pulses, but they purchased greater quantities. This was also the case for overweight participants purchasing LPs. We also found a similar pattern for the oldest age category and ULPs, with a lower probability but greater quantities purchased.

#### 2017 purchases

Some interesting changes were observed in 2017 compared to 2015. Concerning the *probability of purchasing pulse products*, income was no longer significant in 2017, for either LPs or ULPs. As for BMI, moderate obesity was negatively associated with the probability of purchase in 2017 (at the 10% level), at the global level and for LPs. The socioprofessional status of farmer was no longer significant.

Concerning the *quantities* purchased, no income effect was observed at the global level in 2017. However, we still observed—as in 2015—a negative effect of the lower income category (under the poverty line) for LPs. In contrast, this effect was positive for ULPs in 2017. For LPs, county sizes under and above the reference category (2,000–199,999 inhabitants) showed a positive relationship to quantities purchased in 2017.

### The purchase of pulse products during the FAO campaign

**Figure 3:**
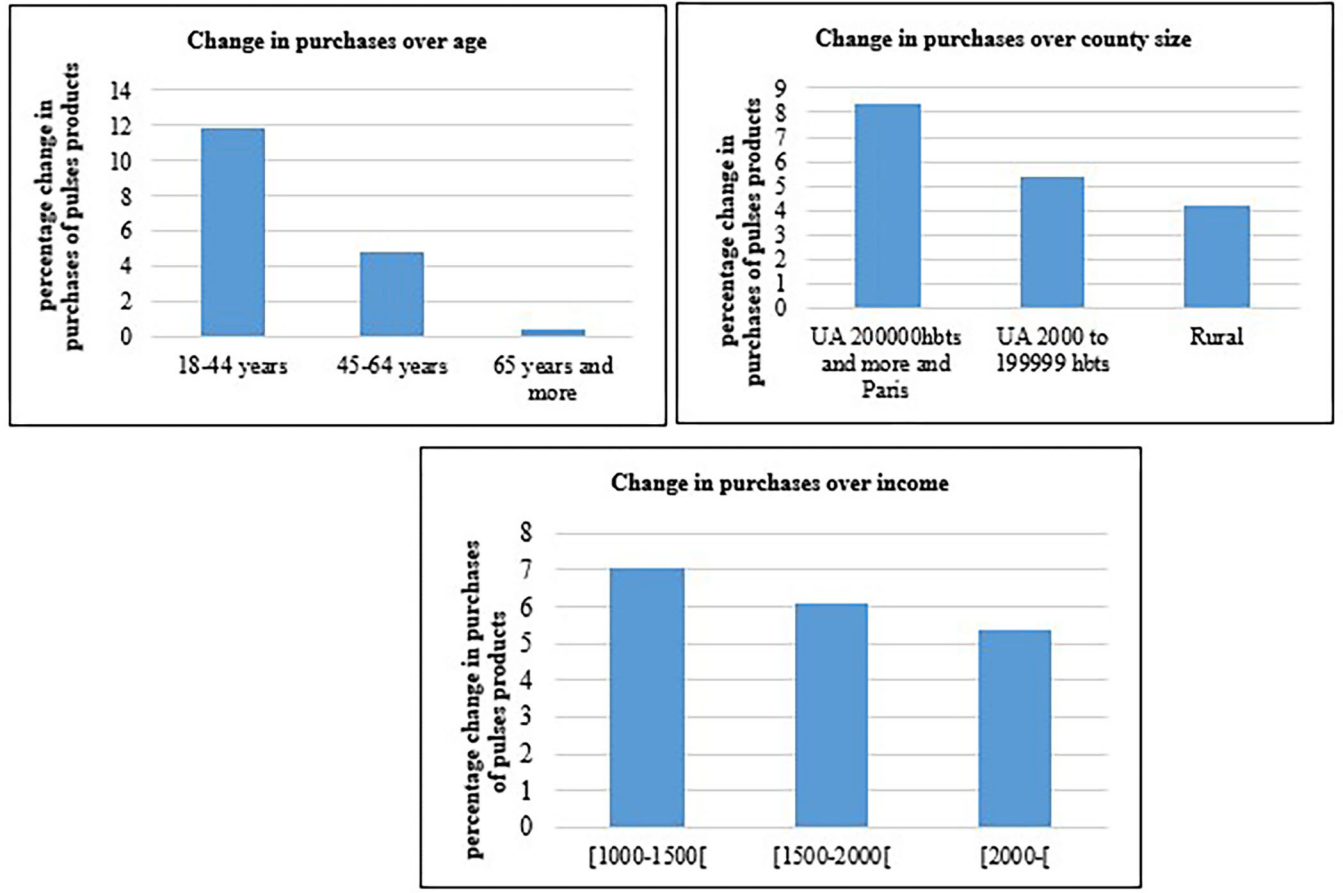
Change (%) in quantities purchased of pulses products over age groups, county size and income classes.

The coefficient associated with price (adjusted unit value) was significantly negative, indicating that a 1% rise in the price was accompanied by a 0.70% decrease in the quantity purchased (Table 3). Controlling for sociodemographic variables, the change in purchasing ρ was significantly positive. Indeed, the purchasing of pulses increased by 8.4%. The Figure 1 shows a steady increase in pulses purchasing since 2014, indicating a gradual preferences change in favor of pulses. For this reason, we estimated a specification that includes a time trend variable. The coefficient associated with this variable is statistically significant and the change in purchasing after the campaign is estimated by 2% (Table 3). Hence, the 8.4% increase observed indicated that there was a gradual preference change in favor of pulses and the impact of the awareness campaign was to boost expenditure on pulses by a further 2%. Our results from the separate two-way fixed-effects model also showed heterogeneous effects observed across various sociodemographic groups (age of the reference person, county size, and household income; cf. Figure 3 and Table 3).

**Table 3:** Changes in purchases of pulses after the FAO awareness campaign.

	**Method 1**	**Method 1 (with time trend)**	**Method 2**	
	**Whole sample**	**Whole sample**	**Sample before information campaign**	**Sample after information campaign**
log(price)	−0.698***	−0.695***	−0.699***	−0.699***
	(0.00118)	(0.0007963)	(0.000683)	(0.000692)
Information campaign (ρ)	0.0815***	0.02027***		
	(0.00109)	(0.001348)		
**Age**				
18–44	−0.0237***	−0.0119***	−0.00554*	0.00262
	(0.00471)	(0.003177)	(0.00288)	(0.00260)
45–64	*Ref*	Ref	*Ref*	*Ref*
65 years +	−0.00137	−0.0160***	−0.00805***	−0.0111***
	(0.00473)	(0.00319)	(0.00282)	(0.00260)
**Monthly income** €**/CU**				
[1,000–1,500]	−0.0229***	−0.01041**	0.00264	−0.0178***
	(0.00487)	(0.00328)	(0.00217)	(0.00269)
[1,500–2,000	−0.00388	0.00030	−0.000720	−0.00257**
	(0.00257)	(0.0017)	(0.00153)	(0.00110)
[2000]	*Ref*	Ref	*Ref*	*Ref*
**Fruit tree owner**				
Yes	0.513***	0.53011***	0.494***	0.503***
	(0.0174)	(0.0117)	(0.00993)	(0.00760)
No	*Ref*	Ref	*Ref*	*Ref*
**Vegetables production at home**				
Yes	−0.198***	−0.2499***	−0.310***	−0.270***
	(0.0160)	(0.0107)	(0.0102)	(0.00787)
No	*Ref*	Ref	*Ref*	*Ref*
**Education level**				
< Post-secondary qualifications	0.0239	−0.0059	−0.0811***	−0.0117
	(0.0219)	(0.0147)	(0.0127)	(0.0110)
Post-secondary qualifications	*Ref*	Ref	*Ref*	*Ref*
1st, 2nd, 3rd year university	−0.0141	0.0156	−0.0381**	−0.0592***
	(0.0258)	(0.0173)	(0.0154)	(0.0136)
Bachelor degree and +	0.0411	0.0325*	0.0653***	−0.0174
	(0.0258)	(0.0173)	(0.0165)	(0.0119)
**Region of residence**				
North	0.0956***	0.1068***	0.158***	0.119***
	(0.0170)	(0.0114)	(0.0101)	(0.00931)
South	*Ref*	Ref	*Ref*	*Ref*
**Body mass index**				
Thinness	−0.0609	−0.071**	−0.0924***	−0.105***
	(0.0430)	(0.0289)	(0.0230)	(0.0182)
Normal weight	*Ref*	Ref	*Ref*	*Ref*
overweight	0.181***	0.097***	−0.0281***	0.0491***
	(0.0177)	(0.0119)	(0.0101)	(0.00902)
Moderate obesity	0.290***	0.1926***	−0.0276**	0.107***
	(0.0243)	(0.0164)	(0.0130)	(0.0127)
Severe and morbid obesity	0.177***	0.1396***	−0.0131	0.0939***
	(0.0320)	(0.0215)	(0.0160)	(0.0177)
No answer	0.103**	0.131***	−0.0429	0.144***
	(0.0492)	(0.0331)	(0.0266)	(0.0209)
Trend		0.0081***		
		(0.00015)		
**Time effects**				
2014T1			*Ref*	
2014T2			−0.0000723	
			(0.000745)	
2014T3			−0.000164	
			(0.000782)	
2014T4			0.000134	
			(0.000767)	
2015T1			0.0445***	
			(0.000750)	
2015T2			0.0444***	
			(0.000764)	
2015T3			0.0443***	
			(0.000823)	
2015T4			0.0447***	
			(0.000795)	
2016 T1				*Ref*
2016T2				−0.0000533
				(0.000683)
2016T3				−0.000163
				(0.000768)
2016T4				0.000119
				(0.000725)
2017T1				0.0429***
				(0.000695)
2017T2				0.0428***
				(0.000708)
2017T3				0.0427***
				(0.000811)
2017T4				0.0429***
				(0.000720)
Constant	−0.213***	−0.2081***	−0.0500***	−0.0694***
	(0.0205)	(0.01378)	(0.0132)	(0.0109)
*N*		2592	1296	1272

Standard errors in parentheses; ^*^p < 0.1, ^**^p < 0.05, ^***^p < 0.01. Models include the sociodemographic variables (age, BMI of the participant), SES (household income, education of the participant), self-consumption availability (orchard, vegetable garden) and localization (region, county size).

ρ: change in purchases before and after the campaign period.

As the dependent variable is the logarithm of the quantity purchased, before interpreting coefficients associated to the information campaign, sociodemographic variables (age, BMI of the participant), SES (household income, education of the participant), self-consumption availability (orchard, vegetable garden) and localization (region, county size), it is important to apply this transformation: 100 x [exp(α_*a*_) - 1] where α_*a*_ is the coefficient associated with variables. For example, the change in purchases before and after the campaign period is 100 x [exp(0.0815) - 1] = 8.4%.

We found that the awareness campaign coincided with an increase in purchases for all sociodemographic groups. The change in quantities purchased over the pre- and post-campaign time period was greater among young people and people living in large urban areas with over 200,000 inhabitants (Figure 3). For instance, the campaign coincided with an 11.8% increase in the purchase of pulse products for participants aged 18–44 years of age, whereas stability was observed for senior participants (65 years and more), with a very small increase estimated at 0.51%. For urban households (200,000 inhabitants and over), an 8.4% increase was observed, vs. 4.2% for rural areas. Regarding income (Figure 3), we found two types of positive effects. The first was overall, and the second involved a stronger effect at the lower income level: 7.1% for the lower-income group and 5.4% in the upper-income group. Regarding the robustness check, the estimations run on the second pseudo-panel sample (59) showed a similar positive effect of ρ, along with a negative association with price (Appendix H).

## Discussion

### Did the profile of pulse consumers change?

Our results show that pulse purchasing was associated with sociodemographic and economic characteristics of households in the pre-campaign and the post-campaign periods. Some effects were comparable between 2015 and 2017, such as the negative effect of younger panelists and the positive effect of older ones on the quantity purchased, in line with the characteristics of an old-fashioned/traditional consumption (61). In 2017, as in 2015, older participants had a lower probability of purchasing ULPs but not LPs. Clearly, older participants did not change their habits and were less attracted to innovative products than younger participants (reference: 45–64 years). In line with the idea of pulses being a traditional food, purchasing is still positively associated with the lowest level of education and negatively with the highest levels of education and higher socioprofessional status.

Interesting differences arose in 2017, indicating a lower importance of sociodemographic variables. In particular, in the “post-campaign” period, pulse product purchasing was not related to BMI or income. Looking at the degree of processing of pulse products, we indeed found that most associations with BMI status seen in 2015 (except for severe/morbid obesity) were not observed in 2017 for LPs. Being thin was also no longer associated with the purchase of ULPs after the campaign. This suggests that before the campaign, LPs were purchased by heavier consumers while ULPs were used by specific consumers, probably those with more plant-based diet patterns, such as vegetarians. In the “post-campaign” period, these patterns were no longer observed. This may be linked to information on the benefits of LPs and ULPs becoming more mainstream as supply developed (36). Accordingly, a larger share of the population purchased these types of products in 2017 (Appendix A).

Our study highlights a complex effect of income. Pulses are traditionally viewed as a low-status food. We observed a positive relationship between the probability of purchase and a lower income in 2015, at the global level and for LPs (this effect disappears in 2017), but a negative association between that income level and the quantity purchased was found in 2015 (and remained in 2017). This means that poorer households buy in total fewer quantities, but they shop more often. Note that Kantar data do not register food-away-from-home. Yet, a French study found that, in the context of choice between dishes meat-based or pulses-based, consumers chose pulses dishes when they were in contexts of ≪ restaurant ≫ or “self-service ≫ rather than in at-home preparation (16)^23^. As higher income households are known to eat more frequently out-of-home than other households, their lower frequency of pulses purchase for at-home-consumption may be more than compensated by their choice of pulses-based dishes when out-of-home ^24^.

Meanwhile, note that for the quantity of ULPs purchased, the association with a lower income level, which was negative in 2015, turned positive in 2017. Therefore, despite increasing prices (Appendix A), the market for processed pulse products appears to have developed even for lower-income households. Since the quantities purchased by all income levels increased (Appendix B), this does not appear to be a pure substitution effect at the expense of raw products but, rather, a real expansion of the market at the household level. Finally, is there a geographical issue? The negative effect of the northern region at the global level and for LPs suggests an influence on consumption of living in areas of production. There could be a self-production effect here, as being a farmer had a negative effect on purchasing in 2015.

Therefore, some differences were observed in 2017 compared to 2015, indicating that changes in behavior occurred between these years. The pure effects of the FAO's information campaign cannot be separated from other factors, however. Besides the change in preferences from the consumer side, some changes regarding supply can also be noted, as illustrated by the different effects observed between LPs and ULPs. Between both years, the variety of products offered in the market increased, and in particular on the consumption innovative ultra-processed preparations (36).

### An increase in purchasing concomitant with the FAO campaign

Our method, which uncovered an increase in pulse purchasing during the FAO campaign, provides an estimated average effect, as in Shankar et al. (40), but this does not imply a causal analysis. Our results may support a positive effect of the FAO campaign, which coincided with an 8.4% increase in the quantity of pulses purchased. Compared with other informational campaigns with positive effects, the FAO campaign's resonance in France appears lower than that of the salt campaign in the UK, which was estimated to have reduced the consumption of salt by 11% (40). The 5-a-day fruit and vegetable campaign in the UK was also found to have a positive effect, since the estimated intake would have been 0.3 portions lower without the campaign (39). Therefore, in France as in other countries, large institutional campaigns do come with and probably enhance positive changes in purchasing behavior.

Recent private initiatives have also been launched to attempt to change people's eating habits, including the “Meatless Monday” campaign, which advocates avoiding meat or fish on Mondays.^25^ However, as for these latter types of information campaign, the positive effect observed after the FAO campaign differed across sociodemographic groups. It was greater for households with younger participants and those living in urban areas, which corresponded to households with lower levels of baseline purchases in 2014 and 2015, before the campaign, thus shortening the relative differences within those population groups (Appendix B). Indeed, younger consumers had been found by Stoll-Kleeman and Schmidt (24) to be more open to flexitarianism, which might help explain this demographic effect.

Concerning income, the greatest increase in pulse purchasing was in lower-income households (1,000–1,500€ monthly). Overall, the increase in purchasing concomitant with the FAO campaign matches changing consumer behavior with the development of the market (36). The anticipation of food manufacturers and their back-and-forth interactions with public policy are well known (68). However, there are still gaps between perceptions of supply and demand (69). Our results suggest a two-tier market, as LP and ULP purchases are clearly differentiated in terms of sociodemographic groups, and in particular with regard to income and BMI. This could mean that both institutional and private strategies—the FAO campaign and market forces—favor a gap between well-informed consumers ready to pay the price for innovative foods, and consumers less focused on pulses, identified as traditional LPs, for reasons of lack of information and price. The launching of these on the market exploits the demand for healthy and sustainable foods and benefits from institutional communication regarding healthy dietary habits. In this context, an encouraging finding is the greater increase in purchasing by young participants and lower-income households, which partly corrects consumption discrepancies between sub-populations and, in particular, income classes. The largest increase in purchasing is registered for the lowest income class (7.1 vs. 5.4% for the highest income class), despite the moderating influence of the rise in prices throughout the period. Such an encouraging effect was not found by Capacci et al. (39) for the fruit and vegetable campaign in the UK, where the 3rd income quartile benefited the most from the campaign effects, indicating a regressive effect in terms of nutritional inequalities. This might be explained by the different profiles of consumers of fruit and vegetables compared to pulses: higher SES in the first case, lower SES in the second case.

Overall, our results suggest that a large institutional campaign may be a useful tool at the global level and also in the perspective of minimizing nutritional inequalities through the reduction of consumption disparities.

### Strengths and limitations

One of the strengths of our study is that it evaluates the change in pulse purchasing before and after a large FAO campaign. Although our method does not allow the identification of a causal effect, the results reveal an increase in purchasing, which has some interesting policy implications (discussed below). In addition, we evaluated the change in pulse purchasing for different subgroups based on income, age, and region and found differential purchasing patterns, in particular with reference to income.

Our study also has some limitations, however. First, the size of the cohorts in our pseudo-panel is restricted (64 households, on average), although we conducted a robustness check by calculating estimates on a sample with larger cohorts (127 households, on average). Second, the length of the period is short (2014–2017), a common drawback shared by other studies (39, 40). Third, Kantar purchasing data are incomplete, as they do not include food-away-from-home and do not represent whole-household consumption. Thus, our evaluation of the increase in pulse purchases during the FAO campaign can only be considered a benchmark.

## Conclusions

Our paper aimed to analyze the change in pulse purchasing before and after an FAO information campaign and discuss the possible effects of this push for awareness. In a first step, we analyzed the influence of sociodemographic characteristics on the purchasing of pulse products. In a second step, we provided an estimation of the change in purchasing patterns.

Then, two main results can be highlighted. First, our results suggest a positive effect of the FAO campaign. There was a gradual preference change in favor of pulses and the impact of the awareness campaign was to boost expenditure on pulses by a further 2%. Our method provides an estimated average effect and not a causal effect, however, similar to the work of Shankar et al. (40). Second, we observed a larger increase in purchasing for low-income households compared to high-income households. We tested the robustness of our results by conducting a similar estimation on a second pseudo-panel sample with larger cohorts and found consistent results.

Our study has certain policy implications. Our results show that there is still a long way to go to reach the level of consumption advised by France's nutritional recommendations (“Programme National Nutrition Santé”). The timing and the extent of the consumer response observed here results both from the FAO design of the campaign and its implementation by local institutions, as well as from market forces that led to greater variety in supply and an increase in prices. Pulse-based products are still considered an old-fashioned food, sometimes perceived as being consumed by those on tight budgets, in spite of efforts to give them a better image in terms of their health benefits. The FAO campaign might have been an initial signal to everyday consumers, as it launched various national initiatives, and this type of awareness raising should be pursued at the level of both public institutions and private stakeholders.

Additional work is needed to build a comprehensive understanding of changes in food purchasing following the campaign. Future work should investigate the potential substitution of animal-protein products by plant-protein ones across a longer timeframe. The results of such work can help support future food policies aimed at improving health and sustainability, as well as the development of the market. Improving communication about the benefits of pulses may help promote this food category, and several channels can be used simultaneously to this end—in particular between the food industry and consumers (69)—as new food market segments develop. Nudging in shopping environments could also favor the purchase of pulses and promote more sustainable consumer behavior.

## Data availability statement

The datasets presented in this article are not readily available because our data come from Kantar Worldpanel, which is a private company, and data users are not at liberty to share the raw data nor any transformed version thereof. Consequently, we do not provide a data appendix. Requests to access the datasets should be directed to france.caillavet@inrae.fr.

## Author contributions

IB, FC, and MA designed the study. IB performed data management and data analysis. FC and IB wrote the first draft of the manuscript. All authors contributed to manuscript revision, read, and approved the submitted version.

## Footnotes

^1^Legumes are soybeans and beans, lentils, chickpeas, and peas. Pulses are defined as dry-harvested leguminous crops, excluding soybeans.

^2^For example, inverse relationships between the consumption of legumes and the risk of cardiovascular disease (5), type 2 diabetes (6), and cardiometabolic risk factors such as overweight/obesity (7) and dyslipidemia (8).

^3^ https://www.dietaryguidelines.gov/sites/default/files/2019-05/2015-2020_Dietary_Guidelines.pdf

^4^ https://www.mangerbouger.fr/Les-recommandations/Augmenter/Les-legumes-secs-lentilles-haricots-pois-chiches-etc

^5^Based on epidemiological studies, consumption recommendations indicate a maximum of < 500 g/week of red meat and < 50 g/day of processed meat (11). French guidelines advise a maximum of 500 g/week and 150 g/week for red meat and processed meat, respectively (12).

^6^On the basis of an average portion of 100 g.

^7^“Étude Individuelle Nationale des Consommations Alimentaires”, based on food recall over three 24-h periods (14). Note that this survey reports information on less-processed pulses (raw, preserved, or frozen products) only and does not include ultra-processed products such as preparations based on pulses.

^8^These, however, consume an average of 52.4 g/day, meeting the 200 g/week recommendation.

^9^Some works rely on balance-sheet data, which only refer to apparent consumption (18).

^10^Particularly influential was a report on red meat consumption and colon cancer (IARC/WHO) published in 2007 (31).

^11^The key slogans to raise awareness were “Pulses are highly nutritious”; “Pulses are economically accessible and contribute to food security at all levels”; “Pulses have important health benefits”; “Pulses foster sustainable agriculture and contribute to climate change mitigation and adaptation”; “Pulses promote biodiversity”. The International Year of Pulses generated 225 events in 63 countries (33).

^12^ http://www.fao.org/pulses-2016/events/fr/?page=30&ipp=5&tx_dynalist_pi1%5bpar%5d=YToxOntzOjE6IkwiO3M6MToiOCI7fQ==

^13^Food-away-from-home was estimated to constitute 20% of all food consumed in 2015 (43) and over 25% of household food expenditure in 2018 (44).

^14^Kantar data make it possible to employ panel data and to follow the same households over multiple years. In our sample, fewer than 60% of households present in 2014 recorded their purchases in 2017; therefore, we did not work with panel data. We considered our databases as cross-sectional and constructed a pseudo-panel in section Estimation of changes in pulse purchasing over the awareness campaign period using a two-way fixed-effects model, which has the advantage of avoiding selection bias associated with attrition effects (which increase with the number of periods) and bias associated with learning effects.

^15^Computed as the ratio of expenditure to quantity.

^16^Similar to Cragg's double-hurdle model, the Heckman model is an alternative method that supposes sequential behavior. In the first step, households decide to participate or not in the market. However, Heckman's model is less flexible. In this model, once consumers pass the participation hurdle they are assumed to have positive consumption, whereas Cragg's model assumes that a potential buyer could have zero consumption. Indeed, Cragg's model postulates that, in addition to an individual being a potential buyer, favorable circumstances have to arise for positive consumption to be observed (i.e., a person is a potential buyer and has sufficient funds to purchase pulses). In other words, in Cragg's model the consumer must pass two hurdles: (1) be a potential buyer of pulses (participation hurdle) and (2) actually buy pulses (consumption hurdle). The first hurdle allows for the possibility that zero consumption is due to non-economic considerations (preferences), while the second hurdle allows for the possibility that zero consumption could be a corner solution.

^17^The Breusch–Pagan test of heteroskedasticity that was carried out led to a rejection of the homoskedasticity assumption (Appendix C).

^18^We included variables that were more likely to cause the heteroskedasticity according to the Breusch–Pagan test. The Breusch–Pagan test consists of regressing the squared residuals of the model on the independent variables. Thus, we were able to identify the variables that were sources of heteroskedasticity.

^19^Here, we deal with statistical cohorts, in contrast to cohorts identified from health surveys. In this paper, a cohort is a group of households with the same characteristics (e.g., “year of birth” of the reference person in the household, or “county size”).

^20^Each quarter is composed of three Kantar four-week periods, with the last quarter including the remaining four periods.

^21^Access to an orchard, access to a vegetable garden, education level, region of residence, and body mass index are all originally dummy variables and became proportional when constructing the pseudo-panel. For example, the region of residence variable comprises 2 levels (North and South), and in the pseudo-panel it indicates the proportion of households in cohort *c* living in the North and in the South.

^22^BMI levels are *thin* ≤ 18.5 kg/M2; *normal weight* < 25 kg/M2; *overweight* < 30 kg/M2; *moderate obesity* < 35 kg/M2; anything above this level is considered *severe obesity*.

^23^One motivation could be saving time of preparation since pulses are perceived as requiring a long preparation (16).

^24^French consumption surveys, INCA3 (14) and CAP Protéines/CREDOC (62) registered pulse consumption, but not income information. Literature on the effect of economic crisis (such as in 2008) which induced an income shock on food consumption, does not bring clear results. Pulses consumption was found to decrease in Greece (63) and in Italy (64), while it increased in France (65), in Spain (66) and in Portugal (67). Besides, it is not clear whether it results from the crisis impact or from the existing trend.

^25^ https://www.lundi-vert.fr/index.php/2020/09/22/les-bienfaits-des-legumineuses/
