# Supplementary material for: Changes in French purchases of pulses during an FAO awareness campaign
Source: Front Nutr. 2023 Jan 26;9:971868. doi: 10.3389/fnut.2022.971868 (PMC9909192; doi:10.3389/fnut.2022.971868)
Supplement: Supplementary file 1 [file Data_Sheet_1.docx]

**APPENDICES ONLINE**

**Appendix A: Evolution of purchases of pulse products (quantities and expenditure) 2014-2017, according to their degree of processing - Purchasers only**

|  | **2014** | | **2015** | | **2016** | | **2017** | |
| --- | --- | --- | --- | --- | --- | --- | --- | --- |
| **Number observations** | 10914 | | 11074 | | 11301 | | 10764 | |
| **All Pulse products** | | | | | | | | |
| **quantities (kg/capita/year)** | 2.68 | | 2.74 | | 2.82 | | 2.86 | |
| **Proportion of purchasers (%)** | 84.94 | | 86.07 | | 87.13 | | 88.41 | |
| **Purchasers Only - Quantities (kg/capita/year)** | 3.15 | | 3.19 | | 3.23 | | 3.24 | |
|  | **Less processed#** | | | | **Ultra-processed#** | | | |
|  | **2014** | **2015** | **2016** | **2017** | **2014** | **2015** | **2016** | **2017** |
| **Proportion of purchasers (%)** | 73.19% | 73.92% | 75.73% | 77.16% | 60.04% | 61.79% | 61.97% | 63.55% |
| **Quantity (kg/year/capita)** | 2.17 | 2.16 | 2.15 | 2.18 | 1.82 | 1.85 | 1.91 | 1.86 |
| **Expenditure (€/year/capita)** | 4.15 | 4.10 | 4.19 | 4.29 | 5.78 | 6.13 | 6.38 | 6.29 |
| **Unadjusted Unit value (€/year/capita)** | 2.11 | 2.06 | 2.14 | 2.17 | 4.24 | 4.38 | 4.65 | 4.83 |
| Source: Kantar 2014-2017; #: Less-processed: unprocessed pulses. preserved and frozen; Ultra-processed: preparations and dishes from pulses | | | | | |  |  |  |

| **Appendix B: Evolution of purchased quantities of pulses according to sociodemographic variables. 2014-2017** | | | | |
| --- | --- | --- | --- | --- |
| **(kg/year/capita)** |  |  |  |  |
|  |  |  |  |  |
|  | **2014** | **2015** | **2016** | **2017** |
| **Household Income ((Monthly income €/CU)** |  |  |  |  |
| < 1000 euros | 2.70 | 2.74 | 2.83 | 2.88 |
| [1000-1500[ | 2.67 | 2.79 | 2.81 | 2.81 |
| [1500-2000[ | 2.57 | 2.70 | 2.68 | 2.78 |
| [2000- [ | 2.77 | 2.74 | 2.93 | 2.99 |
|  |  |  |  |  |
| **Education of the participant** |  |  |  |  |
| <post-secondary qualifications | 3.08 | 3.24 | 3.22 | 3.40 |
| post-secondary qualifications | 2.74 | 2.79 | 2.84 | 2.80 |
| 1^st^, 2^nd^, 3^rd^ year university | 2.39 | 2.46 | 2.55 | 2.54 |
| Bachelor’s degree and + | 2.39 | 2.36 | 2.56 | 2.60 |
|  |  |  |  |  |
| **Age of the participant** |  |  |  |  |
| 18-44 years | 2.28 | 2.29 | 2.36 | 2.36 |
| 45-64 years | 3.07 | 3.15 | 3.18 | 3.26 |
| 65 years and over | 3.17 | 3.40 | 3.40 | 3.46 |
|  |  |  |  |  |
| **County size** |  |  |  |  |
| Rural | 2.69 | 2.73 | 2.84 | 2.83 |
| Urban area 2.000 to 199.999 inhab. | 2.66 | 2.80 | 2.85 | 2.84 |
| Urban area of 200.000 inhab. and over | 2.68 | 2.69 | 2.77 | 2.92 |
|  |  |  |  |  |
| **Region** |  |  |  |  |
| North | 2.64 | 2.69 | 2.79 | 2.83 |
| South | 2.74 | 2.84 | 2.88 | 2.92 |

**Appendix C: Diagnostic and Specification tests (variable: pulse products purchases)**

|  | Test type | Test value | P-value |
| --- | --- | --- | --- |
| Double-hurdle (Cragg) vs Tobit (Year 2015) | LR ^(1)^ | 3257.2558 | 0 |
| Double-hurdle (Cragg) vs Tobit (Year 2017) | LR ^(1)^ | 4038.8276 | 0 |
| Homoscedasticity assumption of residuals (Year 2015) | Breusch -Pagan | 99.266987 | 0 |
| Homoscedasticity assumption of residuals (Year 2017) | Breusch -Pagan | 106.91 | 0 |
| Normality assumption of residuals (Year 2015) | Doornik-Hansen | 10023.198 | 0 |
| Normality assumption of residuals (Year 2017) | Doornik-Hansen | 11064.589 | 0 |

(1) LR test: First, we separately estimated the Tobit model, the double-hurdle model (Probit model that is associated to the participation equation, and the truncated regression model). Then we used the likelihood associated which each model to compute the LR statistic. Following Greene (2000). the LR statistic for this test can be computed as follows:

$$LR= -2[lnL_{T}-\left( lnL_{P}+lnL_{TR} \right)]\sim\chi^{2}(k)$$

where $L_{T}:$likelihood for the Tobit model. $L_{P}:$likelihood for the probit model. $L_{TR}:$likelihood for the truncated regression model and $k$ is the number of independent variables in the equations. When$LR >$ $\chi^{2}(k)$. the test favors the use of the double-hurdle model.

|  |  |  |  |  |
| --- | --- | --- | --- | --- |
| **Appendix D: Associations of sociodemographic and economic variables with purchases of pulse products**  **2015 and 2017**  **Box-Cox Heteroscedastic Double-Hurdle Model** | | | | |
|  | Year 2015 | | Year 2017 | |
|  | Specification 1 | | Specification 1 | |
| VARIABLES | Participation | $Y^{T}$ | Participation | $Y^{T}$ |
| **Age** |  |  |  |  |
| 18-44 | -0.35*** | -0.31*** | -0.23*** | -0.34*** |
|  | (0.036) | (0.027) | (0.037) | (0.027) |
| 45-64 | Ref | Ref | Ref | Ref |
| 65 years + | -0.05 | 0.14*** | -0.05 | 0.12*** |
|  | (0.050) | (0.035) | (0.050) | (0.035) |
| **County Size** |  |  |  |  |
| rural |  | -0.00 |  | 0.04 |
|  |  | (0.029) |  | (0.029) |
| Urban area - from 2000 to 199999 inhabitants |  | Ref |  | Ref |
| Urban area of 200000 inhabitants+ and Paris |  | -0.05* |  | 0.03 |
|  |  | (0.029) |  | (0.030) |
| **Region of residence** |  |  |  |  |
| North | 0.02 | -0.07*** | 0.00 | -0.04* |
|  | (0.032) | (0.025) | (0.033) | (0.025) |
| South | Ref | Ref | Ref | Ref |
| **Monthly income €/CU** |  |  |  |  |
| <Poverty line | 0.12** | -0.13*** | 0.06 | -0.01 |
|  | (0.051) | (0.041) | (0.059) | (0.045) |
| Poverty line to median income | 0.19*** | -0.15*** | 0.07 | -0.02 |
|  | (0.041) | (0.033) | (0.048) | (0.037) |
| Median income to 7th decile | Ref | Ref | Ref | Ref |
| >7th decile | 0.01 | -0.09** | 0.03 | -0.01 |
|  | (0.045) | (0.036) | (0.052) | (0.040) |
| **Socio-professional status** |  |  |  |  |
| Famer | -0.48** | -0.01 | 0.06 | -0.26 |
|  | (0.196) | (0.170) | (0.262) | (0.168) |
| Senior executive | -0.10 | -0.14*** | -0.05 | -0.19*** |
|  | (0.063) | (0.053) | (0.068) | (0.053) |
| Student/Unemployed person | 0.01 | 0.01 | -0.03 | 0.02 |
|  | (0.059) | (0.045) | (0.065) | (0.049) |
| Employee/Manual worker | Ref | Ref | Ref | Ref |
| Associated professionals | -0.00 | -0.13*** | -0.02 | -0.10*** |
|  | (0.036) | (0.028) | (0.038) | (0.028) |
| Liberal profession | 0.03 | -0.04 | -0.09 | -0.06 |
|  | (0.082) | (0.064) | (0.082) | (0.064) |
| **Body Mass Index** |  |  |  |  |
| Thinness | -0.04 | 0.09 | 0.06 | -0.02 |
|  | (0.080) | (0.065) | (0.091) | (0.068) |
| Normal weight | Ref | Ref | Ref | Ref |
|  |  |  |  |  |
| overweight | -0.13*** | 0.03 | -0.06 | 0.00 |
|  | (0.036) | (0.028) | (0.038) | (0.029) |
| Moderate obesity | -0.00 | 0.07 | -0.10* | 0.04 |
|  | (0.053) | (0.040) | (0.052) | (0.040) |
| Severe and morbid obesity | -0.15** | 0.15** | -0.10 | 0.06 |
|  | (0.070) | (0.058) | (0.075) | (0.058) |
| No answer | -0.05 | 0.03 | 0.14 | -0.12 |
|  | (0.109) | (0.086) | (0.131) | (0.089) |
| **Fruit tree owner** |  |  |  |  |
| Yes | 0.15*** |  | 0.15*** |  |
|  | (0.036) |  | (0.037) |  |
| No | Ref |  | Ref |  |
| **Vegetables production at home** |  |  |  |  |
| Yes | 0.13*** |  | 0.11*** |  |
|  | (0.037) |  | (0.038) |  |
| No | Ref |  | Ref |  |
| Constant | 1.13*** | 1.18*** | 1.21*** | 1.05*** |
|  | (0.053) | (0.043) | (0.061) | (0.046) |
| Box-Cox Parameter ($\lambda)$ |  | 0.19*** |  | 0.18*** |
|  |  | (0.009) |  | (0.008) |
| Log likelihood | -24616.55 | | -24209.67 | |
| Observations | 11,074 | 11,074 | 10,764 | 10,764 |

Standard errors in parentheses *** p<0.01, ** p<0.05, * p<0.10.

Specification 1: includes all variables except education variable.

| **Appendix E: Associations of sociodemographic and economic variables with less-processed pulse products purchases (LPs) – Box-Cox Double-hurdle model, 2015 and 2017** | | | | | | | | |  |
| --- | --- | --- | --- | --- | --- | --- | --- | --- | --- |
|  | Year 2015 | | | | Year 2017 | | | |  |
|  | Specification 1 | | Specification 2 | | Specification 1 | | Specification 2 | |  |
| VARIABLES | Participation | $Y^{T}$ | Participation | $Y^{T}$ | Participation | $Y^{T}$ | Participation | $Y^{T}$ |  |
| **Age** |  |  |  |  |  |  |  |  |  |
| 18-44 | -0.29*** | -0.23*** | -0.28*** | -0.19*** | -0.25*** | -0.26*** | -0.24*** | -0.24*** |  |
|  | (0.030) | (0.026) | (0.032) | (0.027) | (0.031) | (0.026) | (0.032) | (0.027) |  |
| 45-64 | Ref | Ref | Ref | Ref | Ref | Ref | Ref | Ref |  |
| 65 years + | 0.02 | 0.20*** | 0.02 | 0.18*** | 0.02 | 0.21*** | 0.01 | 0.20*** |  |
|  | (0.042) | (0.032) | (0.042) | (0.032) | (0.042) | (0.032) | (0.042) | (0.032) |  |
| **County Size** |  |  |  |  |  |  |  |  |  |
| rural |  | -0.03 |  | -0.03 |  | 0.05* |  | 0.05* |  |
|  |  | (0.027) |  | (0.027) |  | (0.027) |  | (0.027) |  |
| Urban area - from 2000 to 199999 inhabitants |  | Ref |  | Ref |  | Ref |  | Ref |  |
| Urban area of 200000 inhabitants+ and Paris |  | -0.01 |  | -0.01 |  | 0.06** |  | 0.06** |  |
|  |  | (0.027) |  | (0.027) |  | (0.028) |  | (0.028) |  |
| **Region of residence** |  |  |  |  |  |  |  |  |  |
| North | -0.07** | -0.08*** | -0.07** | -0.08*** | -0.03 | -0.06*** | -0.04 | -0.06*** |  |
|  | (0.027) | (0.023) | (0.027) | (0.023) | (0.028) | (0.024) | (0.028) | (0.024) |  |
| South | Ref | Ref | Ref | Ref | Ref | Ref | Ref | Ref |  |
| **Monthly income €/CU** |  |  |  |  |  |  |  |  |  |
| <Poverty line | 0.07 | -0.14*** | 0.07* | -0.16*** | -0.00 | -0.11** | 0.00 | -0.11*** |  |
|  | (0.044) | (0.038) | (0.043) | (0.038) | (0.050) | (0.043) | (0.049) | (0.043) |  |
| Poverty line to median income | 0.18*** | -0.14*** | 0.18*** | -0.15*** | 0.03 | -0.04 | 0.03 | -0.04 |  |
|  | (0.036) | (0.031) | (0.036) | (0.030) | (0.041) | (0.035) | (0.041) | (0.035) |  |
| Median income to 7th decile | Ref | Ref | Ref | Ref | Ref | Ref | Ref | Ref |  |
| >7th decile | 0.05 | -0.05 | 0.05 | -0.05 | -0.03 | 0.01 | -0.04 | 0.01 |  |
|  | (0.039) | (0.034) | (0.039) | (0.034) | (0.045) | (0.038) | (0.044) | (0.038) |  |
| **Socio-professional status** |  |  |  |  |  |  |  |  |  |
| Famer | -0.31* | -0.16 |  |  | 0.08 | -0.17 |  |  |  |
|  | (0.181) | (0.160) |  |  | (0.214) | (0.158) |  |  |  |
| Senior executive | -0.18*** | -0.13** |  |  | -0.10* | -0.11** |  |  |  |
|  | (0.055) | (0.051) |  |  | (0.057) | (0.050) |  |  |  |
| Student/Unemployed person | 0.06 | -0.04 |  |  | 0.05 | -0.02 |  |  |  |
|  | (0.051) | (0.041) |  |  | (0.056) | (0.046) |  |  |  |
| Employee/Manual worker | Ref | Ref |  |  | Ref | Ref |  |  |  |
| Associated professionals | -0.03 | -0.07*** |  |  | -0.03 | -0.09*** |  |  |  |
|  | (0.031) | (0.026) |  |  | (0.032) | (0.027) |  |  |  |
| Liberal profession | -0.03 | -0.02 |  |  | -0.03 | 0.01 |  |  |  |
|  | (0.070) | (0.060) |  |  | (0.071) | (0.060) |  |  |  |
| **Body Mass Index** |  |  |  |  |  |  |  |  |  |
| Thinness | -0.01 | 0.06 | -0.00 | 0.06 | -0.02 | -0.06 | -0.02 | -0.06 |  |
|  | (0.070) | (0.061) | (0.070) | (0.061) | (0.075) | (0.065) | (0.075) | (0.064) |  |
| Normal weight | Ref | Ref | Ref | Ref | Ref | Ref | Ref | Ref |  |
| overweight | -0.09*** | 0.07** | -0.10*** | 0.06** | -0.07** | 0.03 | -0.07** | 0.03 |  |
|  | (0.031) | (0.027) | (0.031) | (0.027) | (0.032) | (0.027) | (0.032) | (0.027) |  |
| Moderate obesity | -0.03 | 0.09** | -0.04 | 0.08** | -0.08* | 0.05 | -0.08* | 0.04 |  |
|  | (0.045) | (0.038) | (0.045) | (0.038) | (0.044) | (0.039) | (0.044) | (0.039) |  |
| Severe and morbid obesity | -0.08 | 0.20*** | -0.09 | 0.18*** | -0.08 | 0.11** | -0.08 | 0.11** |  |
|  | (0.062) | (0.053) | (0.062) | (0.053) | (0.065) | (0.052) | (0.065) | (0.052) |  |
| No answer | -0.15 | 0.06 | -0.15 | 0.06 | -0.05 | 0.08 | -0.05 | 0.08 |  |
|  | (0.092) | (0.076) | (0.092) | (0.076) | (0.102) | (0.084) | (0.102) | (0.083) |  |
| **Fruit tree owner** |  |  |  |  |  |  |  |  |  |
| Yes | 0.11*** |  | 0.11*** |  | 0.13*** |  | 0.13*** |  |  |
|  | (0.031) |  | (0.031) |  | (0.031) |  | (0.031) |  |  |
| No | Ref |  | Ref |  | Ref |  | Ref | Ref |  |
| **Vegetables production at home** |  |  |  |  |  |  |  |  |  |
| Yes | 0.17*** |  | 0.17*** |  | 0.15*** |  | 0.14*** |  |  |
|  | (0.031) |  | (0.031) |  | (0.032) |  | (0.032) |  |  |
| No | Ref |  | Ref |  | Ref |  | Ref | Ref |  |
| **Education level** |  |  |  |  |  |  |  |  |  |
| < Post-secondary qualifications |  |  | 0.01 | 0.05 |  |  | 0.05 | 0.06* |  |
|  |  |  | (0.037) | (0.031) |  |  | (0.038) | (0.031) |  |
| Post-secondary qualifications | Ref |  | Ref | Ref |  |  | Ref | Ref |  |
| 1st , 2nd ,3rd year university |  |  | -0.02 | -0.13*** |  |  | 0.02 | -0.11*** |  |
|  |  |  | (0.037) | (0.032) |  |  | (0.038) | (0.032) |  |
| Bachelor’s degree and + |  |  | -0.10*** | -0.10*** |  |  | -0.05 | -0.03 |  |
|  |  |  | (0.039) | (0.034) |  |  | (0.040) | (0.034) |  |
| Constant | 0.69*** | 0.61*** | 0.69*** | 0.60*** | 0.82*** | 0.49*** | 0.79*** | 0.46*** |  |
|  | (0.045) | (0.039) | (0.048) | (0.042) | (0.052) | (0.044) | (0.054) | (0.045) |  |
| Box-Cox Parameter ($\lambda)$ |  | 0.09*** |  | 0.09*** |  | 0.09*** |  | 0.09*** |  |
|  |  | (0.010) |  | (0.010) |  | (0.010) |  | (0.010) |  |
| Log likelihood | -20224.33 | | -20215.44 | | -19980.18 | | -19973.8 | |  |
| Observations | 11,074 | 11,074 | 11,074 | 11,074 | 10,764 | 10,764 | 10,764 | 10,764 |  |

Standard errors in parentheses *** p<0.01, ** p<0.05, * p<0.10.

Less-processed products (LPs) include raw, preserved and frozen pulses products.

Specification 1: includes all variables except education variable; Specification 2: includes all variables except Socio-professional status variable

| **Appendix F: Associations of sociodemographic and economic variables with ultra-processed pulse products purchases (ULPs)– Box-Cox Double-hurdle model, 2015 and 2017** | | | | | | | | |
| --- | --- | --- | --- | --- | --- | --- | --- | --- |
|  | Year 2015 | | | | Year 2017 | | | |
|  | Specification 1 | | Specification 2 | | Specification 1 | | Specification 2 | |
| VARIABLES | Participation | $Y^{T}$ | Participation | $Y^{T}$ | Participation | $Y^{T}$ | Participation | $Y^{T}$ |
| Age |  |  |  |  |  |  |  |  |
| 18-44 | -0.19*** | -0.21*** | -0.17*** | -0.16*** | -0.14*** | -0.25*** | -0.12*** | -0.21*** |
|  | (0.028) | (0.034) | (0.029) | (0.036) | (0.028) | (0.034) | (0.029) | (0.036) |
| 45-64 | Ref | Ref | Ref | Ref | Ref | Ref | Ref | Ref |
| 65 years + | -0.21*** | 0.14*** | -0.22*** | 0.11** | -0.29*** | 0.11** | -0.30*** | 0.08* |
|  | (0.038) | (0.044) | (0.038) | (0.044) | (0.037) | (0.046) | (0.037) | (0.046) |
| County Size |  |  |  |  |  |  |  |  |
| rural |  | -0.01 |  | -0.01 |  | 0.01 |  | 0.00 |
|  |  | (0.037) |  | (0.036) |  | (0.037) |  | (0.037) |
| Urban area - from 2000 to 199999 inhabitants |  | Ref |  | Ref |  | Ref |  | Ref |
| Urban area of 200000 inhabitants+ and Paris |  | -0.11*** |  | -0.10*** |  | -0.04 |  | -0.04 |
|  |  | (0.037) |  | (0.037) |  | (0.038) |  | (0.038) |
| Region of residence |  |  |  |  |  |  |  |  |
| North | 0.02 | 0.03 | 0.02 | 0.03 | 0.04 | 0.01 | 0.04 | 0.01 |
|  | (0.026) | (0.031) | (0.026) | (0.031) | (0.026) | (0.032) | (0.026) | (0.032) |
| South | Ref | Ref | Ref | Ref | Ref | Ref | Ref | Ref |
| Monthly income €/CU |  |  |  |  |  |  |  |  |
| <Poverty line | 0.07 | -0.10** | 0.07 | -0.12** | 0.05 | 0.15** | 0.03 | 0.13** |
|  | (0.041) | (0.050) | (0.040) | (0.049) | (0.046) | (0.058) | (0.046) | (0.057) |
| Poverty line to median income | 0.10*** | -0.21*** | 0.09*** | -0.22*** | 0.02 | 0.04 | 0.01 | 0.03 |
|  | (0.033) | (0.041) | (0.033) | (0.041) | (0.037) | (0.047) | (0.037) | (0.047) |
| Median income to 7th decile | Ref | Ref | Ref | Ref | Ref | Ref | Ref | Ref |
| >7th decile | 0.01 | -0.16*** | 0.02 | -0.15*** | -0.01 | 0.04 | -0.01 | 0.04 |
|  | (0.037) | (0.046) | (0.037) | (0.045) | (0.041) | (0.053) | (0.041) | (0.052) |
| Socio-professional status |  |  |  |  |  |  |  |  |
| Famer | -0.23 | 0.15 |  |  | -0.41** | 0.16 |  |  |
|  | (0.172) | (0.218) |  |  | (0.177) | (0.249) |  |  |
| Senior executive | -0.04 | -0.07 |  |  | -0.03 | -0.21*** |  |  |
|  | (0.053) | (0.067) |  |  | (0.054) | (0.069) |  |  |
| Student/Unemployed person | 0.05 | -0.00 |  |  | -0.00 | -0.00 |  |  |
|  | (0.047) | (0.055) |  |  | (0.051) | (0.062) |  |  |
| Employee/Manual worker | Ref | Ref |  |  | Ref | Ref |  |  |
| Associated professionals | -0.03 | -0.15*** |  |  | -0.02 | -0.11*** |  |  |
|  | (0.029) | (0.036) |  |  | (0.029) | (0.036) |  |  |
| Liberal profession | 0.07 | -0.11 |  |  | -0.08 | -0.14* |  |  |
|  | (0.066) | (0.079) |  |  | (0.065) | (0.083) |  |  |
| Body Mass Index |  |  |  |  |  |  |  |  |
| Thinness | -0.11* | 0.17** | -0.11 | 0.18** | 0.02 | 0.03 | 0.02 | 0.03 |
|  | (0.066) | (0.084) | (0.066) | (0.084) | (0.069) | (0.089) | (0.069) | (0.090) |
| Normal weight | Ref | Ref | Ref | Ref | Ref | Ref | Ref | Ref |
|  |  |  |  |  |  |  |  |  |
| overweight | -0.06* | 0.01 | -0.06** | 0.01 | -0.03 | -0.00 | -0.03 | -0.01 |
|  | (0.029) | (0.036) | (0.029) | (0.036) | (0.029) | (0.037) | (0.029) | (0.037) |
| Moderate obesity | -0.02 | 0.06 | -0.02 | 0.04 | -0.04 | 0.05 | -0.04 | 0.04 |
|  | (0.042) | (0.048) | (0.042) | (0.048) | (0.041) | (0.050) | (0.041) | (0.050) |
| Severe and morbid obesity | -0.01 | 0.05 | -0.02 | 0.04 | -0.02 | 0.02 | -0.02 | 0.01 |
|  | (0.059) | (0.067) | (0.059) | (0.067) | (0.060) | (0.074) | (0.060) | (0.074) |
| No answer | 0.05 | 0.05 | 0.05 | 0.05 | -0.03 | -0.14 | -0.03 | -0.13 |
|  | (0.089) | (0.101) | (0.089) | (0.099) | (0.094) | (0.109) | (0.094) | (0.109) |
| Fruit tree owner |  |  |  |  |  |  |  |  |
| Yes | 0.09*** |  | 0.09*** |  | 0.04 |  | 0.04 |  |
|  | (0.029) |  | (0.029) |  | (0.029) |  | (0.029) |  |
| No | Ref |  | Ref |  | Ref |  | Ref |  |
| Vegetables production at home |  |  |  |  |  |  |  |  |
| Yes | 0.09*** |  | 0.09*** |  | 0.02 |  | 0.02 |  |
|  | (0.029) |  | (0.029) |  | (0.029) |  | (0.029) |  |
| No | Ref |  | Ref |  | Ref |  | Ref | Ref |
| Education level |  |  |  |  |  |  |  |  |
| < Post-secondary qualifications |  |  | -0.02 | 0.18*** |  |  | 0.11*** | 0.07 |
|  |  |  | (0.034) | (0.040) |  |  | (0.035) | (0.042) |
| Post-secondary qualifications | Ref |  | Ref | Ref |  |  | Ref | Ref |
| 1st , 2nd ,3rd year university |  |  | -0.06 | -0.01 |  |  | 0.04 | -0.11** |
|  |  |  | (0.035) | (0.043) |  |  | (0.035) | (0.044) |
| Bachelor’s degree and + |  |  | -0.10*** | -0.10** |  |  | 0.02 | -0.18*** |
|  |  |  | (0.037) | (0.046) |  |  | (0.037) | (0.048) |
| Constant | 0.32*** | 0.26*** | 0.35*** | 0.17*** | 0.42*** | 0.06 | 0.37*** | 0.05 |
|  | (0.042) | (0.052) | (0.045) | (0.055) | (0.048) | (0.059) | (0.049) | (0.061) |
| Box-Cox Parameter ($\lambda)$ |  | 0.08*** |  | 0.07*** |  | 0.08*** |  | 0.08*** |
|  |  | (0.009) |  | (0.009) |  | (0.008) |  | (0.008) |
| Log likelihood | -17966.5 | | -17951.539 | | -17681.7 | | -17669.75 | |
| Observations | 11,074 | 11,074 | 11,074 | 11,074 | 10,764 | 10,764 | 10,764 | 10,764 |

Standard errors in parentheses *** p<0.01. ** p<0.05. * p<0.10.

Ultra-processed pulses products (ULPs) include preparations and ready-to-eat dishes from pluses.

Specification 1: includes all variables except education variable; Specification 2: includes all variables except Socio-professional status variable.

| Appendix G: Change in purchases of pulse products (%) by sociodemographic groups |
| --- |

**Appendix H: Estimation on two pseudo-panel for robustness check**

|  |  |  |
| --- | --- | --- |
|  | Pseudo-panel 1 **^a^** | Pseudo-panel 2 **^b^** |
| log(price) | -0.698*** | -0.310*** |
|  | (0.00118) | (0.000658) |
| $\rho$ ^c^ | 0.0815*** | 0.0606*** |
|  | (0.00109) | (0.00124) |

Dependent variable: log (pulses products purchases); Standard errors are reported in parentheses. The pre-campaign period includes 2014 and 2015 (8 quarters) and the post-campaign period includes 2016 and 2017 (8 quarters).

^a^ Pseudo-panel 1

We defined our cohorts using two variables: the “year of birth” of the reference person in the household and the “county size” composed of three modalities (rural area under 2,000 inhabitants, urban area - from 2,000 to 199,999 inhabitants, urban area of 200,000 inhabitants and over). Thereby we constituted 165 annual cohorts observed at minimum over 8 quarters and maximum over 16 quarters (4 years*4 quarters). Our pseudo-panel includes 2,592 observations (study sample).

^b^ Pseudo-panel 2

Contrary to the previous pseudo-panel, this one is composed of 84 biannual cohorts observed over 8 quarters and maximum over 16 quarters. It includes 1,320 observations. Each cohort including on average 127 individuals. These cohorts were similarly constructed based on “year of birth” and “county size” variables.

The first three cohorts comprise households born in 1935-1936 and living respectively in rural, urban area from 2000 to 199,999 inhabitants, urban area of 200,000 inhabitants and over. The last three cohorts comprise households born in 1989-1990 and living respectively in rural, urban area from 2,000 to 199,999 inhabitants, urban area of 200,000 inhabitants and more.

^c^ $\rho$ represents the change in purchases before and after the campaign period.
